# Fu’s subcutaneous needling for chronic gastritis with observations on serum gastric function indices: A case report

**DOI:** 10.1097/MD.0000000000043438

**Published:** 2025-07-18

**Authors:** Juanjuan Shi, Ziyue Zhang, Jin Lu, Xiang Liu, Lina Fei, Hongyi Yu

**Affiliations:** a Department of Acupuncture, Nanjing Hospital of Traditional Chinese Medicine, Nanjing, China; b Faculty of Chinese Medicine, Macau University of Science and Technology, Macau, China; c Endoscopy Center, Nanjing Hospital of Traditional Chinese Medicine, Nanjing, China; d Clinical Laboratory, Nanjing Hospital of Traditional Chinese Medicine, Nanjing, China; e Department of Pharmaceutical Product Management and Marketing, Belvarosi Gyogyoszertar (Downtown Pharmacy), Szervita tér, Hungary.

**Keywords:** acupuncture, case report, chronic gastritis, Fu’s subcutaneous needling, serum gastric function

## Abstract

**Rationale:**

Fu’s subcutaneous needling (FSN) is an innovative acupuncture therapy derived from traditional techniques. It employs disposable floating needles and auxiliary needle guides to conduct a broad, swaying motion. The therapy aims to enhance blood perfusion and oxygen supply to local muscles, activate the body’s self-healing mechanisms, and ultimately promote drug-free recovery. This case study examines FSN’s application in treating chronic gastritis (CG) and and its effects on serum gastric function indices.

**Patient concerns:**

A 44-year-old female patient presented with complaints of epigastric discomfort, nonspecific pain or a burning sensation, along with occasional belching, anorexia, nausea, and vomiting. Additionally, the patient reported poor sleep quality and the presence of formless stools.

**Diagnoses:**

The patient was diagnosed with CG by gastroscopy.

**Interventions:**

FSN was administered 3 times/wk. No adverse or unanticipated events were observed during the treatment period. The entire treatment course spanned a duration of 4 weeks.

**Outcomes:**

Following the treatment, the patient reported the absence of significant discomfort in her epigastrium, and noted the resolution of both vague pain and burning sensation. Additionally, she experienced an increase in appetite, regular bowel movements, and improved sleep quality.

**Lessons:**

FSN has been demonstrated to significantly alleviate epigastric discomfort associated with CG and to have a positive impact on appetite, sleep quality, and bowel movements. Moreover, FSN demonstrates a significant and sustained improvement in serum gastric function indices, with no observed relapse. However, due to the limitations inherent in a single clinical observation case, further randomized controlled clinical trials are warranted.

## 1. Introduction

Chronic gastritis (CG) represents a chronic inflammatory condition affecting the gastric mucosa. It can be triggered by a variety of pathological factors, such as Helicobacter pylori (Hp) infection, duodenal and bile reflux, prolonged use of nonsteroidal anti-inflammatory drugs, improper diet, and psychological stress, leading to long-term damage to the gastric mucosal barrier and its protective functions. CG lacks specific clinical manifestations, and some patients may remain asymptomatic during the early stages. Common clinical syndromes encompass persistent and recurrent epigastric pain, abdominal distension, early satiety, postprandial fullness, belching, sour regurgitation, nausea, and vomiting, along with dyspeptic symptoms that are associated with emotional factors.

In accordance with the Correa’s cascade, if the disease is not treated promptly, a stepwise progression of lesions in the gastric mucosa can be observed as patients age, culminating in intestinal-type gastric carcinoma. Therefore, the diagnostic strategy for CG is aimed not just at fixing the presence of gastric mucosal inflammation, but also at gastric cancer (GC) risk stratification in a particular patient.^[1]^ Chronic atrophic gastritis (CAG), commonly regarded as the precancerous diseases of the stomach, is a critical stage in the multistep process of carcinogenesis. Its prevalence is positively correlated with the incidence of GC. CAG primarily manifests as inflammatory infiltration, glandular atrophy, partial intestinal metaplasia (IM) and dysplasia, and appears to be asymptomatic and reversible.

The serum gastric function test detection for pepsinogen type I and II (PG I and PG II), PG I/PG II ratio, gastrin-17 (G-17) can help to determine the extent of gastric mucosal atrophy. It is an effective and noninvasive method for CAG screening, and has a role in the setting of GC prevention.^[2]^ Pepsinogen (PG) is synthesized by the stomach exclusively and includes 2 subtypes, PG I and PG II, and dynamically change in the development of the CAG. Consequently, PG can be used as a diagnostic indicator for gastric mucosal lesions, with high sensitivity and specificity.^[3]^ PG I/PG II ratio reflects the atrophy of the gastric mucosa. As gastric mucosal atrophy progresses, the level of PG I and PG I/PG II ratio are significantly reduced. Variations in G-17 levels reflect the severity of lesions in the epithelial cells of the gastric mucosa. A decrease in gastric acid secretion and an elevation in G-17 levels indicate the presence of a more severe lesion of the gastric mucosal epithelial cells.

The exact mechanism of CG pathogenesis is complex and unclear. General treatment primarily involves symptomatic management, which includes anti-Hp therapy, acid suppression therapy, and the administration of gastric mucosal protective agents to mitigate the pathological factors contributing to the disease. Although it can alleviate irritation and damage to the gastric mucosa, the underlying glandular lesions within the gastric mucosa cannot be fundamentally resolved. Inevitably, every treatment modality has its limitations. For instance, the adverse drug reactions associated with antibiotics, recurrent attacks, and other significant side effects must be considered. The need for a more effective and less invasive treatment option has led to the exploration of alternative therapies.

Nowadays, traditional acupuncture has been widely applied for CG treatment as a complementary and alternative therapy. Fu’s subcutaneous needling (FSN) is a modern acupuncture therapy that represents an innovation upon traditional acupuncture techniques. Practitioners identify the optimal entry point by palpating the tightened muscles and then proceed to insert the needle into the subcutaneous tissue using an auxiliary needle guide. The therapy involves the simultaneous execution of a swaying movement and a reperfusion approach on the tightened muscles, liberating them from the pathological states characterized by cold, stiffness, tension, and slippage. As the affected muscles are progressively restored to a state of softness and warmth, the diseases can be significantly ameliorated.

Our study illustrates that FSN can not only noticeably relieve the clinical symptoms of patients with CG and optimize the serum biochemical indices of gastric mucosal lesions, but also can reverse or even prevent the gastric mucosal IM to a certain extent.

In this study, we describe a pioneering clinical case involving a 44-year-old female patient who was diagnosed with CG. Upon the application of FSN therapy, the patient experienced a significant alleviation of her clinical symptoms, and her serum biochemical markers indicative of gastric function normalized, leading to a stable and enduring improvement in her condition. To our knowledge, this represents the inaugural documented instance where FSN has been employed to treat CG, with its therapeutic efficacy rigorously assessed through the monitoring of serum gastric function biochemistry. This case underscores the potential of FSN as a novel treatment modality for CG and warrants further investigation into its clinical applications.

## 2. Case presentation

In April 2024, a 44-year-old female patient diagnosed with CG was treated at Nanjing Hospital of Chinese Medicine. The patient presented with complaints of epigastric discomfort, nonspecific pain, and a burning sensation, along with occasional belching, anorexia, nausea, and vomiting. Additionally, she reported poor sleep quality and the presence of formless stools. Given these symptoms, a comprehensive evaluation was conducted, including a gastroscopy and serum gastric function detection, to further elucidate the nature and extent of her gastric condition.

The first gastroscopy report, issued on June 6, 2023, by the Jiangsu Provincial Hospital of Chinese Medicine, reveals the following diagnoses:

1. CG with erosions and hyperplasia.
2. Cardia inflammation: inflammation of the cardia.
3. Hp negative.
4. Mild chronic non-atrophic gastritis.
5. Local superficial gastric mucosal lesion with IM.

The first serum gastric function test results, issued on April 11, 2024, by Nanjing Hospital of Chinese Medicine, are as follows:

- Gastric mucosal health marker (G-17): 46.43 pmol/L.
- PG I: 261.56 μg/L.
- PG II: 22.45 μg/L.
- PG I to PG II ratio: 11.65.
- Vitamin B_12_: 932 pmol/L.
- Folic acid: 9.3 nmol/L.

Initially, the patient expressed a preference for acupuncture treatment. However, following consultation, she elected to undergo FSN therapy.

**Figure 1.**
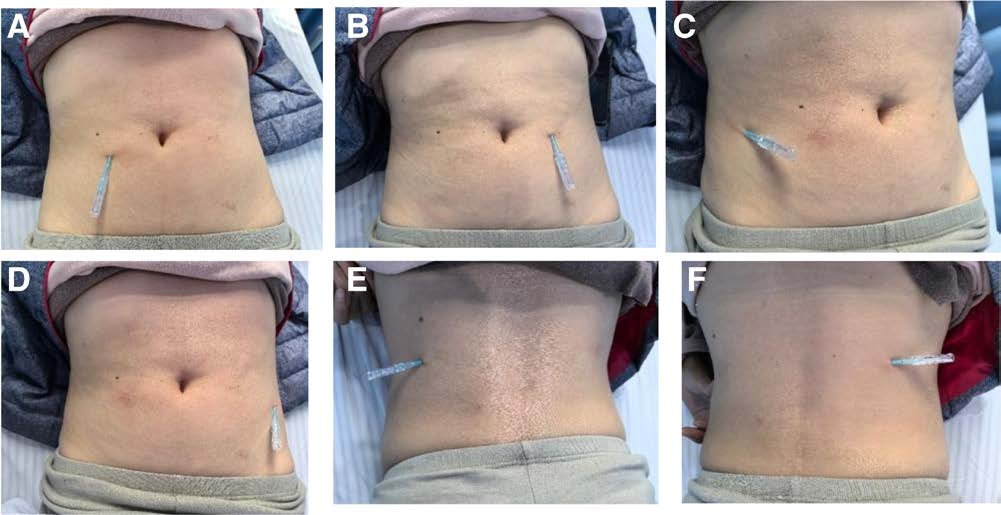
The Fu’s subcutaneous needling insertion points were selected in the upper middle section of the bilateral rectus abdominis muscle (A, B) bilateral obliques abdominis (C, D), and bilateral middle section of erector spinae muscles (E, F).

Upon examination, we identified abnormal tension in the bilateral rectus abdominis, bilateral oblique abdominis, and bilateral middle section of the erector spinae muscles. The insertion points were selected in the upper middle section of the bilateral rectus abdominis muscles (Fig. 1A, B), bilateral oblique abdominis muscles (Fig. 1C, D), and bilateral middle section of the erector spinae muscles (Fig. 1E, F). The FSN needle, designed and patented in China (patent number: CN97114318, Nanjing FSN Medical Appliances Co., Ltd., China), was utilized.

**Figure 2.**
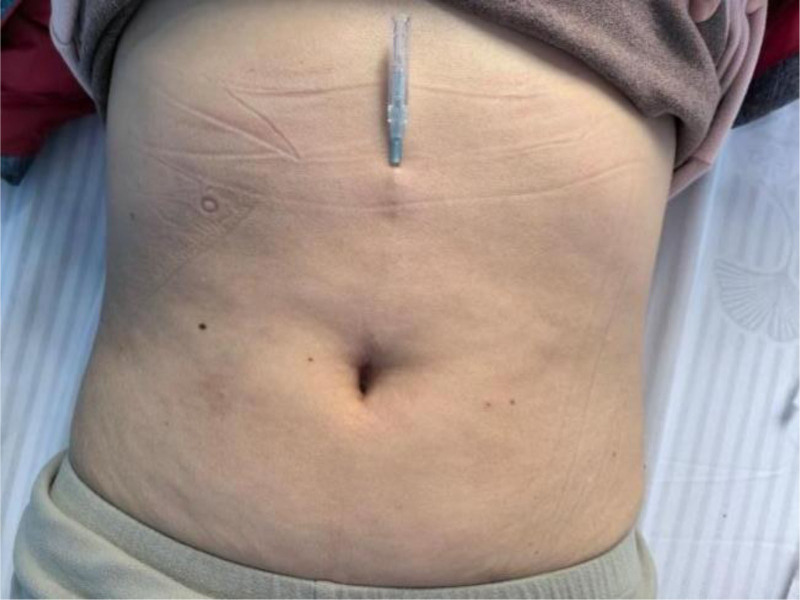
The tube was left in place at Zhongwan (CV12) for a period of 4 h.

The skin was disinfected with povidone-iodine. The disposable floating needle was placed within the auxiliary needle guide and subsequently introduced into the subcutaneous tissue. Upon complete insertion, the needle tip was retracted to the superficial subcutaneous fascia layer and then advanced horizontally. Once the needle was fully inserted, the tip was withdrawn into the tube, and the slot on the needle tool was secured to prevent damage to the soft tissues. Subsequently, the needle body was subjected to fan-shaped horizontal dispersion movements at an angle of 30° and a frequency of approximately 180 oscillations/min. Each entry point was swept and dispersed for a duration of 2 minutes, following which the needle was retained in place for an additional 10 minutes. The uniform operation was reiterated for every subsequent entry point. Ultimately, the needle was withdrawn, and the tube was left in place at Zhongwan (CV12) for a period of 4 hours (Fig. 2). The treatment protocol was maintained for a duration of 4 weeks, with sessions conducted 3 times/wk.

Upon the culmination of the 4-week therapeutic protocol, the patient reported the absence of significant discomfort in her epigastrium, and noted the resolution of both Nonspecific pain and burning sensation. Additionally, she experienced an increase in appetite, regularity in bowel movements, and an improvement in sleep quality. Throughout the therapeutic intervention, there were no recorded instances of adverse or unforeseen events.

The gastroscopy report following FSN treatment, issued on June 13, 2024, by Nanjing Hospital of Chinese Medicine, presented the following findings:

1. CG without pain;
2. Multiple polypi in the stomach were removed via cold snare polypectomy;
3. Hp tested negative;
4. Mild to moderate chronic superficial inflammation of the antrum was observed.

Posttreatment serum gastric function test report was issued on May 31, 2024 by Nanjing Hospital of Chinese Medicine:

- Gastric mucosal health marker (G-17): 1.5 pmol/L.
- PG I: 101.74 μg/L.
- PG II: 5.93 μg/L.
- PG I to PG II ratio: 17.16.
- Vitamin B_12_: 769 pmol/L.
- Folic acid: 5.2 nmol/L.

Follow-up assessments of the intervention process and posttreatment respectively conducted at 1-month intervals revealed that the patient reported no significant discomfort in the epigastric region.

The reexamination of serum gastric function detection was issued on July 3, 2024 by Nanjing Hospital of Chinese Medicine:

- Gastric mucosal health marker (G-17): 1.78 pmol/L.
- PG I: 134.28 μg/L.
- PG II: 7.53 μg/L.
- PG I to PG II ratio: 17.83.
- Vitamin B_12_: 1073 pmol/L.
- Folic acid: 4.4 nmol/L.

A marked improvement can be observed in patient’s subsequent gastroscopy report, without the presence of erosion and IM in gastric mucosa. Following a 1-month course of FSN treatment, her serum biochemical indices of G-17, PG II and vitamin B_12_ returned to normal levels. The serum biochemistry tests reviewed 1 month after the cessation of treatment reported that G-17 and PG II were maintained, whereas vitamin B_12_ was elevated to the abnormal range.

Following completion of the initial treatment course, the patient reported a satisfactory health status. To maintain therapeutic benefits, the aforementioned FSN therapy has been administered on a biweekly basis and is ongoing at the present time. Consequently, all associated symptoms have been resolved. The patient did not receive concurrent medical treatment during the whole treatment period.

The clinical evaluation of serum gastric function in this case is displayed in Table 1.

**Table 1:** Serum gastric function evaluation of FSN treatment.

Indices	Before treatments	After treatments	Reexamination	Normal value
G-17	46.43	1.5	1.78	1.02–7.05
PG I	261.56	101.74	134.28	>70
PG II	22.45	5.93	7.53	3–20
PG I/PG II ratio	11.65	17.16	17.83	>3.0
Vitamin B_12_	932	769	1073	187–883
Folic acid	9.3	5.2	4.4	3.5–19.5

FSN = Fu’s subcutaneous needling, G-17 = gastrin-17, PG I = pepsinogen I, PG II = pepsinogen II.

**Figure 3.**
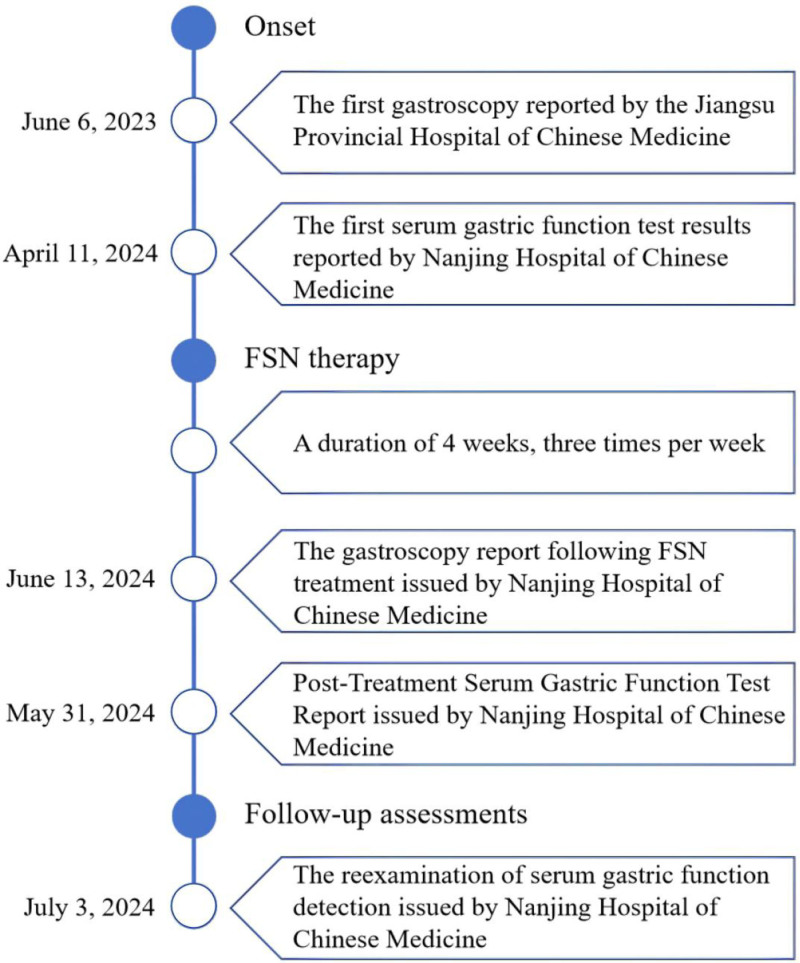
Timeline of this case. FSN = Fu’s subcutaneous needling.

The timeline of this case is shown in Figure 3.

## 3. Discussion

From the perspective of FSN, CG is classified as a disease directly caused by tightened muscles themselves^.[4]^

The gastric smooth muscle, under the influence of diverse etiological stimuli, exhibits excessive contractions and is chronically subjected to conditions of ischemia and hypoxia, culminating in the development of pathologic muscular tissue. Furthermore, this condition is associated with the presence of tension in the abdominal muscles. The patient exhibits pathological tension in the rectus abdominis, obliquus abdominis, and erector spinae muscles. The persistent contraction of these muscles manifests a deleterious effect on the gastric region. The stomach endures compressive forces from the surrounding muscular structures, which may further impair the blood vessels therein. Consequently, this compression leads to a reduction in blood flow and oxygen supply to the aforementioned region, thereby exacerbating the hypoxic-ischemic spasms of the gastric smooth muscle.

This muscular tension and its impact on the stomach’s autorhythmicity are crucial to understanding the symptoms of CG. The normal autorhythmicity of the gastric smooth muscle serves as the physiological foundation for gastric digestion and the subsequent propulsion of food into the duodenum. This autorhythmicity can be compromised by hypoxemia, resulting in abnormalities of the gastric antral contractions and the process of gastric emptying. Consequently, an excessive accumulation of food and gastric acid within the stomach may ultimately occur. The protracted retention of an excessive amount of food within the gastrointestinal tract can engender a spectrum of symptoms, including abdominal distention, eructation, and a diminished appetite in affected individuals. Furthermore, the excessive accumulation of gastric acid may precipitate conditions such as acid reflux, pyrosis, and potentially lead to the erosion of the gastric mucosa. Simultaneously, muscular spasms in the region surrounding the stomach can induce an elevation in intra-abdominal pressure, which in turn can precipitate symptoms of abdominal pain, nausea, and emesis.

FSN presents an innovative therapeutic approach that targets muscle dysfunction to alleviate these symptoms.The potential underlying mechanisms of the therapeutic effects of FSN in this case can be delineated as follows: By addressing the hypertonicity of the abdominal musculature, FSN facilitates the relaxation of the gastric smooth muscle and the musculature in the surrounding affected areas. Consequently, this intervention mitigates the compressive forces exerted upon the vasculature. With the resumption of blood perfusion and oxygen supply, the symptoms associated with CG can be significantly alleviated. Zhongwan (CV12), designated as the front-mu point of the stomach, is prominently featured among the acupoints commonly selected in the traditional acupuncture treatment of CG.^[5]^ Emerging research has evidenced that acupuncture at CV12 can result in a substantial amelioration of the condition of CAG.^[6]^ As a logical corollary, the tube positioned at CV12 is conjectured to augment therapeutic efficacy by delivering a sustained stimulus, thereby enhancing the curative outcome.

The progression of CG and its treatment outcomes are closely monitored through biochemical indices. CG is a process that gradually extends from the superficial surface of the gastric mucosa to the deeper to the glandular region, followed by destruction, reduction and even atrophy of the glands. This case represents that a marked improvement in the serum biochemical indices correlating with gastric mucosal lesions was observed in the patient after the administration of FSN therapy. This observation implies that FSN is capable of enhancing the reparative and regenerative capabilities of the gastric mucosa that has been adversely affected. Furthermore, it indicates that FSN possesses the potential to reverse or hinder the advancement of gastric mucosal IM during its incipient and mild phases, to a moderate degree. Following the conclusion of the treatment period, the normalization of the primary indicators of serum gastric function, which spans a duration of 1 month, is indicative of its stable therapeutic effect.

The impact of CG on patients’ quality of life and mental health is significant, making effective treatment crucial. The recurring and persistent symptoms of CG severely affect the quality of life of patients, which may pose a serious challenge to their mental health. Within the context of this clinical report, FSN has illustrated a potential role in promoting the reparative and regenerative processes of the gastric mucosa that has been adversely affected. This is accomplished through the relaxation of hypertensive muscles and the enhancement of blood and oxygen circulation within the stomach, thus providing a secure, facile, and protractedly efficacious therapeutic intervention for the palliative care of CG.

## 4. Conclusion

The assessment of FSN’s efficacy in treating CG is an ongoing area of research. Furthermore, the existing efficacy evaluation system for FSN therapy in the context of CG is deficient in its observation of biochemical indicators, such as pepsinogen and serum gastrin levels. This case represents the inaugural report detailing the therapeutic impact of FSN on CG, with its therapeutic efficacy being assessed through the observation of biochemical indices related to serum gastric function. Our methodological approach directly addresses these existing gaps in clinical assessment.

## Acknowledgments

We are grateful to the patient for her participation in the study and allowing for publication of this case report. We thank Xiang Liu, Lina Fei for their excellent technical assistance, and Jin Lu for the technical consultation.

## Author contributions

**Investigation:** Xiang Liu, Lina Fei.

**Supervision:** Jin Lu.

**Writing – original draft:** Juanjuan Shi, Ziyue Zhang.

**Writing – review & editing:** Hongyi Yu.
